# Gender Equity and Decent Work in the Health Workforce in Nepal: Insights From Community Health Volunteers, Nurses, and Doctors

**DOI:** 10.1111/nhs.70445

**Published:** 2026-11-05

**Authors:** Christine Bigler, Sony K.C., Susan Thieme

**Affiliations:** ^1^ Interdisciplinary Center for Gender Studies University of Bern, Switzerland Bern Switzerland; ^2^ Kathmandu University Lalitpur Nepal; ^3^ Institut for Geography University of Bern Bern Switzerland

**Keywords:** decent work, gender equity, health workforce, Nepal, nursing

## Abstract

Gender equity in the health workforce is crucial for achieving the Sustainable Development Goals. However, persistent inequities undermine workers' well‐being, service delivery, and health system sustainability. This study examines how gender norms and hierarchical structures shape working conditions across Nepal's healthcare workforce. Drawing on 66 qualitative interviews and three stakeholder meetings with Female Community Health Volunteers (FCHVs), nurses, and doctors working in decentralized and centralized settings, we demonstrate how feminization, undervaluation, and the care–cure divide produce inequities in recognition, pay, autonomy, and career progression. FCHVs provide essential community health services for minimal remuneration. Nurses face high workloads and constrained advancement, and women physicians encounter gendered barriers to leadership and decision‐making. Reconceptualizing decent work as a continuum ranging from unpaid to professional roles, we argue that health workforce equity is fundamental to broader health equity by shaping service quality, equitable access, and sustainable care. Gender‐transformative and equity‐oriented labor policies are required to strengthen health systems and advance SDG 3 (health), SDG 5 (gender equality), and SDG 8 (decent work).

## Key Points

- Gender norms and job segregation shape conditions for nurses, doctors, and volunteers, undermining staff well‐being and the quality and equity of care.
- Decent work is a continuum from unpaid community roles to professional posts; feminized “care” roles remain undervalued, underpaid, and overlooked.
- What to do in practice: pay and formally recognize community health work; ensure fair pay, safe staffing, and career pathways for nurses, and promote women's leadership to advance SDGs 3, 5, and 8.

## 1 Introduction

Gender^1^ is one of the most significant social determinants of health and healthcare (Heise et al. 2019). Achieving gender equity in the health workforce is fundamental to building a sustainable health system (Manandhar et al. 2018; World Health Organization [WHO] 2020). Advancing gender equity as a pathway to gender equality^2^ is essential for the Sustainable Development Goals, particularly SDG 3 on health, SDG 5 on gender equality, and SDG 8 on decent work. Women constitute the majority of health workers and contribute substantially to these goals through care provision and health promotion (United Nations [UN] 2015; WHO 2020, 2023).

Gender equality, the focus of SDG 5, is also a cross‐cutting objective of the 2030 Agenda. Gender inequality, rooted in hierarchical power relations, continues to shape health systems and undermine well‐being. Prevailing power disparities between women and men continue to perpetuate gendered roles and the division of labor (Heymann et al. 2019; Stern et al. 2018). To date, no country, including Nepal, has fully achieved gender equality (World Economic Forum [WEF] 2022).

Health workers make a pivotal contribution to the 2030 Agenda, as healthy communities underpin social and economic progress (Agyeman‐Manu et al. 2023; WHO 2023). Ensuring decent working conditions for health workers is therefore a prerequisite for sustainable improvements in health and development (Rowe et al. 2018). SDG 8 aims to achieve full and productive employment and decent work for all, including equal pay for work of equal value (Dossey et al. 2019; UN 2015). As early as 1989, Vandana Shiva argued that dominant development models, grounded in Western patriarchal ideals, are unsustainable as they rely on the exploitation of nature and women, including unpaid and invisible labor (Mies and Shiva 2016; Oswald Spring 2020; Shiva 1989). From this perspective, decent work is central to sustainability because it challenges systems that obscure or devalue care work by valuing all forms of labor that sustain social and ecological well‐being (Chigbu and Nekhwevha 2023; Eloi 2021).

Despite its importance, research on decent work remains limited, especially in low‐ and middle‐income countries (Pereira et al. 2019). From a feminist perspective, prevailing definitions are incomplete because they privilege paid employment while overlooking the unpaid reproductive and care work that women disproportionately perform alongside paid work (Bidegain Ponte and Rodriguez Enriquez 2016; Doss 2011). Globally, women undertake approximately three times as much unpaid care work as men, sustaining households and communities (WHO 2023).

Recognizing the significance of care delivered by people without extensive formal training is critical. Much of this work occurs at the household and community levels without remuneration and is vital for accessing reproductive, maternal, and child health services, as well as for preventing and treating infectious and noncommunicable diseases (Langer et al. 2015; Maher and Cometto 2016). In Nepal, the work of approximately 50 000 Female Community Health Volunteers (FHCVs) is a prime example of this. Working voluntarily, they make vital contributions to primary healthcare and have been instrumental in improving public health outcomes. However, they often receive limited recognition and compensation (Adhikari et al. 2022). Similar patterns of feminized workforces, low pay and reliance on voluntary labor are evident across South Asia, positioning Nepal within a broader regional landscape of gendered inequalities in health systems (Joshi et al. 2024).

Against this global and regional backdrop, Nepal provides a compelling case for examining how gender norms, power relations and labor dynamics shape the health workforce. This study analyses the situation of healthcare workers in Nepal through the lenses of gender and decent work, identifying the structural and social factors that support or hinder sustainable working environments. The central research question is: what role do gender equity and decent work play in creating a sustainable work environment for healthcare workers in Nepal?

## 2 Healthcare Workers From a Gender Perspective

Women's growing participation in paid work, driven by industrialization and globalization, has been described as the feminization of the labor market (Kabeer and Natali 2013; Tejani and Milberg 2016). This trend is coupled with greater labor segmentation, informal employment and persistent gender income gaps (Kabeer 2012; Perrons 2021; Razavi et al. 2012). At the same time, women continue to bear the primary responsibility for unpaid reproductive and domestic labor, creating a double workload (de Brauw et al. 2014; Doss 2011; Rock and Périlleux 2023).

This is what Chant (2014) refers to as the feminization of responsibilities and obligations. These processes are sustained by horizontal segregation, whereby women and men perform distinct types of work, and vertical segregation, whereby women encounter barriers to professional advancement. Both reinforce occupational hierarchies and limit access to power, income, and status. Norms defining men's and women's work influence both individual choices and institutional structures, preventing women from gaining access to education and property and securing decent employment and quality job progression (Bigler 2022; Hay et al. 2019).

This pattern is also apparent in health systems, which reflect and reinforce existing social and gender inequalities and norms (Gupta et al. 2019). An estimated 67% of health and social care workers are women, a share that continues to rise (Shannon et al. 2019; WHO 2023). Gendered socialization leads women to take on care roles such as nursing, midwifery, and frontline community work. Meanwhile, men are more likely to occupy cure roles such as physicians and specialists. In South Asia, the feminization of care is particularly visible: Accredited Social Health Activists in India, Lady Health Workers in Pakistan and Anganwadi workers, as well as FCHVs in Nepal, are all women providing essential health services (Adhikari et al. 2022; Joshi et al. 2024). These occupational patterns are reinforced by gender norms that sustain a division between care and cure (Folbre 2017; Hay et al. 2019; WHO 2023).

Nursing and midwifery are highly feminized professions. Globally, 85% of nurses are women, and nursing represents the largest professional group in the healthcare sector at 59% (WHO 2020). By contrast, physician roles are associated with greater autonomy, remuneration, and prestige (Wingfield 2017). However, a higher share of female doctors does not ensure equal treatment, pay, or opportunities (Hay et al. 2019; Langer et al. 2015). Evidence shows that, as feminization increases, physician wages have stagnated or even declined (Jena et al. 2016; Ramakrishnan et al. 2014). Furthermore, the medical profession operates through a strict hierarchy (Peadon et al. 2020). Medical students and residents occupy the lowest levels, relying on supervisors for clinical direction and career advancement, which depends on positive evaluations from those in authority (Apostol et al. 2025; Walton 2006).

Across global health systems, gendered horizontal and vertical segregation persists and remains a key driver of inequity (WHO 2019). Despite accounting for around 70% of the workforce, women hold only around 25% of senior positions, and only two United Nations health‐related agencies are led by women. Limited authority constrains women's ability to influence systems and their own careers, contributing to stress, dissatisfaction and burnout (Gupta et al. 2019; Hay et al. 2019). The health sector also exhibits a larger gender pay gap than most others and workers face further decent work challenges, including long hours and exposure to violence (WHO 2020). These disadvantages are experienced differently at the intersection of gender with other social categories, such as age, class and ethnicity, so an intersectional perspective is crucial (Crenshaw 1989; Gupta et al. 2019).

Gender‐responsive approaches recognize and aim to redress unequal norms and roles, while gender‐transformative approaches additionally target the gender pay gap and gender‐based violence and promote women's leadership (Bigler et al. 2024; Boniol et al. 2019). Focusing on gender equity and power relations is thus crucial for strengthening decent work and sustainability in the health sector, as well as for creating systems that challenge structural hierarchies rather than reproducing them (Hay et al. 2019).

## 3 Methods

This study adopted an iterative, reflexive qualitative design (Breuer et al. 2019), comprising sequential phases that built on one another and informed the focus of the next (Figure 1). Phase I (September–December 2022) was an exploratory pilot involving a broad range of health system actors, through which three professions central to Nepal's health system (medical doctors, nurses, and FCHVs) were identified as the analytical focus. Three subsequent phases deepened the engagement: Phase II (August–September 2023), shaped by nationwide health‐sector protests, focused on doctors and nurses; Phase III (January 2024) on FCHVs through photovoice; and Phase IV (March–June 2025) on nurses. Phase V (September 2025) brought the three professions together for three stakeholder meetings. The five phases were analyzed together as a single, integrated dataset rather than as separate studies. This sequential design was itself analytically motivated: rather than examining each profession in isolation, it traced how gender structures the health workforce (Shannon et al. 2019; WHO 2019).

**FIGURE 1:**
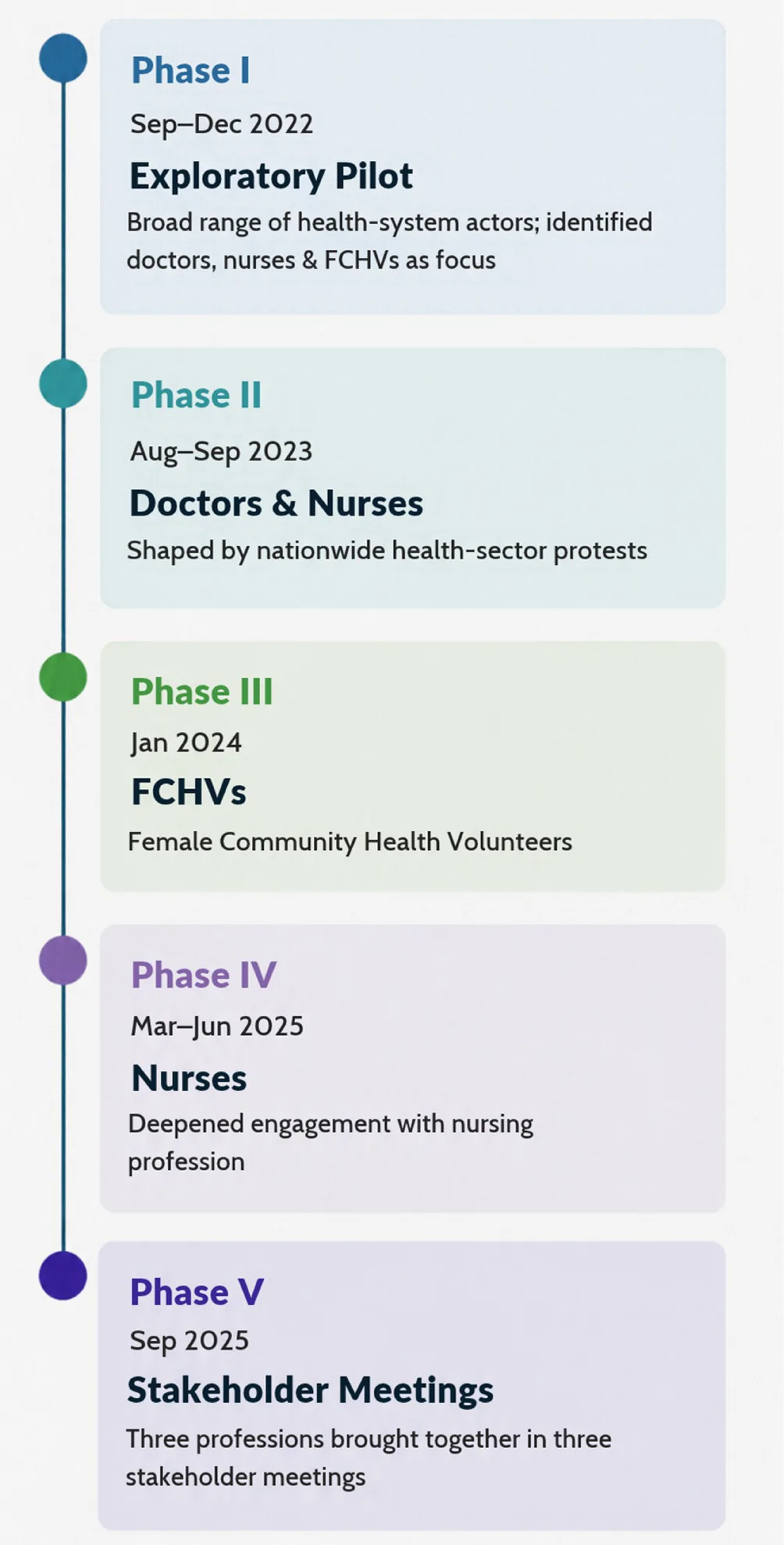
Data collection phases (Phases I–V).

Grounded in the feminist framework set out above, which understands economic and health systems as dependent on women's unpaid and invisible labor, and thus as systematically devaluing feminized care work (Chant 2014; Mies and Shiva 2016; Oswald Spring 2020), the study treated health workers' situated knowledge as a legitimate basis for understanding. This motivated a participatory visual approach with the FCHVs, who interpreted their own realities (Wang and Burris 1997).

### 3.1 Setting, Recruitment and Participants

Purposive sampling was used throughout, adapted to each phase: with snowball sampling in the exploratory pilot (Phase I); during the health‐sector protests (Phase II); at ward level for FCHVs in Lalitpur and Palung (Phase III) and for nurses (Phase IV); and, in Phase V, to draw in stakeholders from the three identified professions. Recruitment was mostly by telephone and through the wards for the FCHVs. For the doctors, we sought a gender‐balanced sample to include women's and men's perspectives. Table 1 shows the number of women, men and both genders interviewed per health profession, mirroring the gendered care/cure hierarchy: exclusively women among the unpaid FCHVs; predominantly women in nursing; and all male in policy and management.

**TABLE 1:** Interviews by professional group and gender (Phases I–IV).

Professional group	Women	Men	Women and men	Total interviews
FCHV	19	0	0	19
Nurses	18	1	1	20
Medical doctors and residents	9	7	1	17
Policymakers, managers, and officials	0	4	0	4
Other (educators, psychologists, NGO)	2	3	1	6
Total	48	15	3	66

In Phase V, three stakeholder meetings were held, one with each professional group. The doctors' meeting comprised 14 women and five men; the nurses' meeting comprised 18 participants, of whom one was a man; and the FCHV meeting comprised 21 participants, including three men.

### 3.2 Data Collection

The study was a Swiss–Nepali research partnership. Phases I, III, and V were conducted jointly, and Phases II and IV by Nepali researchers. A semi‐structured guide of narrative questions, follow‐up prompts, and a topic checklist was used across Phases I–IV and adapted per participant group. Sampling proceeded iteratively until few new insights emerged, approaching theoretical saturation (Breuer et al. 2019). During the Phase V meetings, findings from Phases I–IV were discussed with all groups, and policy recommendations were co‐developed with participants rather than derived by the team alone.

### 3.3 Data Analysis

All interviews and the three stakeholder meetings were audio‐recorded, transcribed, and analyzed following a grounded‐theory approach (Breuer et al. 2019; Strauss and Corbin 1996). Coding combined inductive and deductive strategies: inductive coding revealed unanticipated themes, while deductive coding drew on the study's feminist framework and the literature to develop theoretical sensitivity and initial open codes. Analysis was iterative, with data generation and analysis in parallel (Kekeya 2016; Merriam 1998). Through axial coding, open codes were consolidated into the selective categories shown in Table 2, which were then triangulated in the stakeholder meetings.

**TABLE 2:** From open to selective coding and triangulation during stakeholder meetings.

Open coding (Phase I–IV)	Selective coding (Phase I–IV)	Triangulation during stakeholder meeting (Phase V)
Gender; Caste; Age; Location; Work place; FCHV; Bachelor nurse; Diploma nurse; Medical doctors/Medical officer; Nursing assistant; Paramedic; Public perception/stigma of health workers; Duties; Door to door; Division of labor in the household; Women's and men's roles; Gender equality; Paid and unpaid (care) work; Work–family balance; Family support and reaction; Motivation to enter nursing	Feminization and the care–cure divide	Gender equity in care delivery
Ward duty; Payment; Challenges with payment; Satisfaction with payment; Amount of FCHVs per ward; Working hours; Working days per month/week; Workload; Patient numbers; Salary structures; Co‐worker relationships; Recognition; Protest/strikes; Pandemic/COVID allowance; Workplace safety and violence; Migration/brain drain	Devaluation of feminized care work	Fair work, fair pay and social protection across all staff categories
Training; Education/school level; Skills training; Awareness of the profession; Prior knowledge and orientation; Career development and opportunities; Professional development; Leadership and management; Recognized diseases; Mental health at ward level; Training in mental health; Future development and future plans; Changes expected; Retention; Advice for future FCHVs and nurses	Gendered access to training and advancement	Training aligned with service needs and equitable access

### 3.4 Ethical Considerations

The study adhered to recognized research‐ethics standards and the institution's principles of good scientific practice. As it examined working conditions, gender and unpaid labor rather than health‐related personal data, it fell outside the scope of the applicable national research act, and the competent regional ethics committee declined jurisdiction. As a competent institutional ethics committee was only established in January 2026, formal approval was neither legally required nor available. Participation was voluntary and based on ongoing written consent, which in the photovoice phase also covered taking and using images of identifiable persons. Confidentiality was protected through pseudonymization and secure storage, and attention was paid throughout to the power asymmetries of cross‐cultural research and to reciprocity with participants.

### 3.5 Rigor and Reflexivity

The study's quality was assessed against established criteria for grounded theory and qualitative research (Breuer et al. 2019; Flick 2019). Empirical grounding rested on constant comparison across the coding stages, with categories grounded in the data yet informed by theoretical sensitivity (Strauss and Corbin 1996). Sampling, data generation and transcription decisions were transparently documented, and rigor was further supported by partial second coding, team interpretation, and communicative validation with participants in Phase V. Reflexivity was integral to quality: the team comprised Nepali and Swiss‐based researchers with backgrounds in nursing, development studies, gender studies and geography, differently positioned by nationality, language and institutional power. Following reflexive grounded theory and feminist standpoint theory (Breuer et al. 2019; Harding 2008), they reflected on how these positions and the power relations shaping knowledge production affected access, interpretation and the research relationship, treating an explicit standpoint as a strength rather than a weakness.

## 4 Results

This section is organized into three sub‐sections focusing on the gendered organization of work across the health system: FCHVs, nurses, and medical doctors. Within each sub‐section, we bring together evidence from decentralized ward‐level settings and centralized district and tertiary hospitals, highlighting how gender norms structure roles, responsibilities, and power.

### 4.1 Female Community Health Volunteers

FCHVs play a pivotal role in Nepal's primary healthcare system, serving as the foundation of community‐based health services. As the backbone of the Nepali health system, they are responsible for conducting household visits, identifying individuals in need of medical attention, and ensuring timely referrals to health posts or hospitals. Their duties span household visits, referrals, health check‐ups for vulnerable groups, immunization, health education, and providing basic medication. The following two photographs were taken by two study participants, illustrating their work at the health post and in the neighborhood (Figures 2 and 3).

**FIGURE 2:**
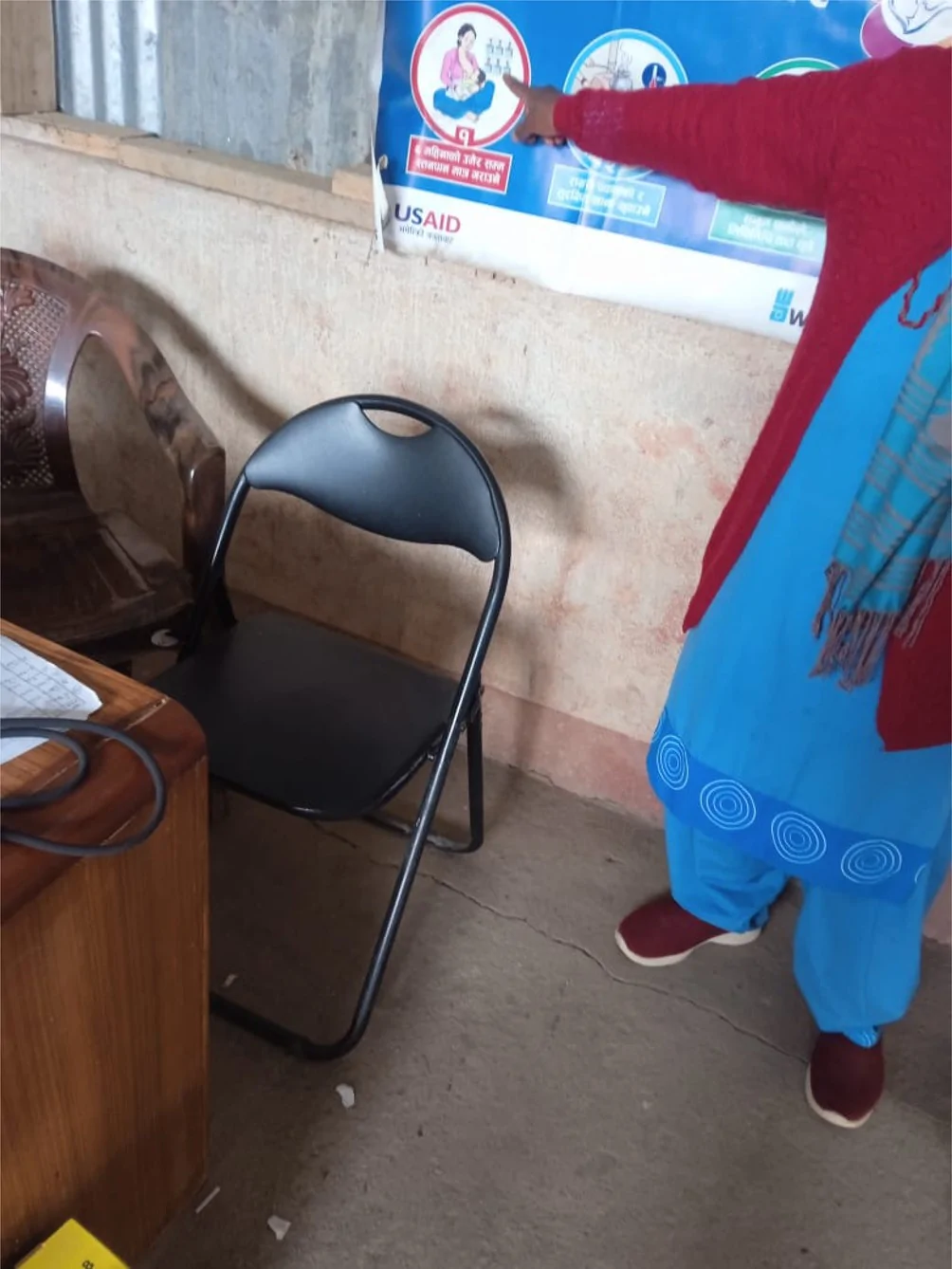
An FCHV providing health education at a health post, Palung. Photograph taken by a study participant.

**FIGURE 3:**
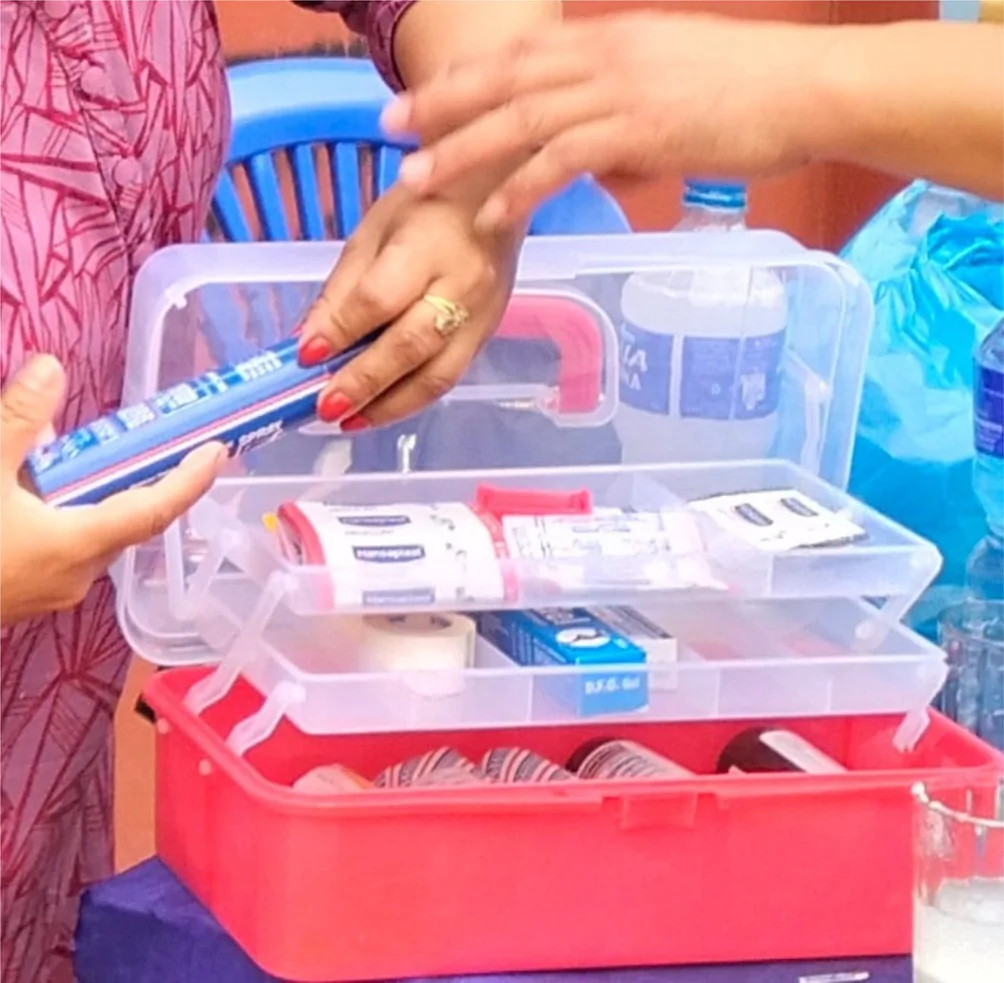
An FCHV's medical kit, used to care for patients in the neighborhood, Lalitpur. Photograph taken by a study participant.

FCHVs are especially important in rural areas, where they often serve as the only link between households and the formal health system. As one NGO representative observed:

*These female health volunteers are overburdened*.

Kiran, NGO representative, Kathmandu

While the role of FCHVs is highly respected and often sought‐after within communities, it remains voluntary and heavily gendered. Being married is widely regarded as a prerequisite for FCHVs, who are typically married women balancing professional and domestic responsibilities. Many described difficulties managing their duties alongside family obligations. One FCHV with over 30 years of experience recalled:

*I was a daughter‐in‐law who would go out to work. Because of that, the community judged me. Now people respect me, but when I started, it was really tough*.

Ganga, FCHV, Lalitpur

This testimony reflects the social negotiations women must undertake to gain acceptance in public roles traditionally reserved for men, or in unremunerated community service.

Despite their vital contribution, FCHVs lack formal medical education and only receive periodic training in areas such as diabetes management, vitamin A supplementation, polio prevention, nutrition, and ear, nose, and throat care. Many expressed a need for more frequent and additional training, particularly in mental health, to handle the emerging challenges within their communities.

*We need frequent training to understand new diseases, but we don't really know enough. Sometimes when people come to ask questions, we don't have the knowledge to share*.

Babita, FCHV, Lalitpur

The feminization of the FCHV cadre is clear in its membership and work organization. FCHVs described a gendered division of labor in which men, who are uncommon in the cadre, are typically assigned back‐office or logistical functions such as stock management and scheduling, while women undertake door‐to‐door care provision. This work primarily serves women, particularly during pregnancy and childbirth, reinforcing the association between women and caregiving.

Although indispensable, FCHVs' remuneration is minimal and irregular. Most receive no regular salary, only a modest monthly stipend of typically around 1000–1500 NPR (approximately 7–11 USD), often disbursed as a lump sum only once or twice a year, together with occasional allowances for uniforms or transport. In rural areas, some work a fixed number of days, typically around two per week, for a small honorarium, whereas in urban areas arrangements vary by ward budget and payments are often delayed, so that volunteers routinely cover costs such as travel themselves. This underpayment, together with the voluntary framing of the role, reflects the feminization and devaluation of care, whereby women's contributions are treated as natural extensions of domestic work rather than recognized as professional labor.

Despite these challenges, FCHVs express pride in their work and acknowledge its positive impact on maternal and child health. Their collaboration with nurses and health posts has contributed to declining infant mortality rates and improved vaccination coverage. However, their lack of recognition and fair pay underscores a persistent gender hierarchy within the health system, where women's essential labor is undervalued, informalized, and rendered invisible.

### 4.2 Nurses

Nurses occupy a central position within Nepal's healthcare system, linking primary, district, and tertiary levels of care. The profession is highly feminized, reflecting deep‐rooted gender norms that associate care work with women's social roles. As one interviewee mentioned, only four male nurses are active in Nepal. While nurses are essential to the delivery of quality healthcare, their contributions are frequently undervalued and shaped by gendered hierarchies that position them below doctors regarding decision‐making authority.

At the decentralized ward level, diploma nurses staff health posts and small community facilities, offering outpatient care, immunizations, maternal and child health services, and preventive care. They coordinate closely with FCHVs, who identify cases in the community, while nurses provide treatment and ensure referrals and follow‐ups. In rural settings, where doctors are scarce, diploma nurses often act autonomously, assuming both clinical and managerial responsibilities, as the following quote shows:

*In the rural context, it is quite different as there are no senior doctors above the junior nurse. So, she has the autonomy to give everything*.

Sunita, nursing student, Kathmandu

Nurses at this level report improvements in maternal and infant health, which they attribute to growing awareness, education, and collaboration with FCHVs. As one diploma nurse noted:

*If a woman stops coming for check‐ups, we call her, and if we cannot reach her, the FCHVs go to her home. The volunteers play a really important role*.

Rupa, diploma nurse, Lalitpur

Yet workloads remain high, with 15 to 20 patients seen daily. Despite their commitment, these nurses face limited resources, long hours, and low pay, illustrating how care work is feminized and undervalued.

In centralized district and tertiary hospitals, diploma and bachelor‐level nurses, also known as staff nurses, provide inpatient and outpatient care. They perform critical clinical tasks such as monitoring patients, administering medication, and supporting recovery. However, their role is constrained by rigid hierarchies dominated by doctors and administrators. The following quote from a former nurse reflects this:

*As a psychologist, I can decide, but as a nurse, I had to consult for even minor decisions. One nurse cared for around twenty patients; it was overwhelming*.

Akriti, former nurse, Kathmandu

Nurses reported apparent differences between rural and urban hospitals. In rural facilities, staff shortages mean nurses have greater autonomy, whereas in urban centers, they must closely follow doctors' instructions. Many young nurses, particularly bachelor‐level graduates, expressed frustration about career stagnation, low salaries, limited recognition and minimal decision‐making power:

*Even if you complete your bachelor's or master's, you are still seen as a diploma nurse. People think nurses just follow doctors' orders*.

Aya, nursing student, Kathmandu

Pay disparities are stark. Private hospitals offer as little as 12 000 NPR (90 USD) per month, compared to around 20 000 NPR (150 USD) in public institutions. Taking on unpaid “volunteer” work before securing a salaried post is common. These conditions, combined with hierarchical discrimination and limited professional growth, prompt many nurses to consider migrating abroad (e.g., Poudel et al. 2018; Prakash 2018). Nursing remains framed as a caring and self‐sacrificing profession, and nurses are often expected to display constant politeness and emotional composure.

*We were expected to do everything […] even if something happened to us, they wouldn't care. No one said ‘Take rest.’ We just had to keep going*.

Soraya, nurse, Kathmandu

Public stereotypes can label assertiveness as rudeness. However, a generational shift is visible. Younger nurses are increasingly speaking up about mental health, workload, and workplace inequities, reflecting both resistance and a transformation in professional identity. Nurses' experiences in both decentralized and centralized settings highlight persistent gender inequalities: essential labor that is undervalued, restricted autonomy, and contributions constrained by hierarchical and patriarchal norms.

### 4.3 Medical Doctors

District and tertiary hospitals in Nepal serve as both teaching institutions and service providers. They host interns, residents in specialist training, and senior consultants, creating a workforce with varied qualifications and responsibilities. Role and rank determine working hours and pay, and most doctors rotate between inpatient and outpatient services. Hospitals operate through clear hierarchies. New patients are typically assessed initially by residents, who carry primary responsibility for initial management and present cases to supervising specialists.

Residents balance demanding clinical schedules with preparation for examinations. Reported salaries range from approximately 20 000 to 42 000 NPR (140–300 USD) per month, depending on the hospital. Long shifts and inadequate rest contribute to stress and burnout. As one final‐year medical doctor explained:

*In some countries, residents are prevented from working more than 80 hours. In Nepal during the COVID crisis, some doctors stayed in the hospital for up to a week. This highlights severe burnout, especially for young doctors*.

Sushant, medical doctor, Kathmandu

He also expressed cautious optimism about later career stages:

*The older the doctor, the better their life is. Senior doctors are specialists, and their duties are more limited, so quality of life increases*.

Sushant, medical doctor, Kathmandu

Aspirations to migrate are widespread (Phuyal 2024). Many students seek residency or employment abroad in pursuit of better salaries, facilities and professional recognition. A senior clinician summarized this trend:

*Over 50 per cent want to relocate to the United States, Canada or Australia. In some colleges, 80 to 90 per cent of undergraduates aspire to opportunities abroad, so very few want to remain*.

Bishnu, head of the medical department, Kathmandu

Those who complete their specialization overseas seldom return, contributing to brain drain.

Gender dynamics shape medical careers. While historically most physicians were men, respondents described an emerging cohort of women medical students, with an approximate female‐to‐male ratio of 40% to 60% among current cohorts.^3^ Segregation by speciality persists. Orthopedics remains male‐dominated, often justified by assumptions about physical endurance and long hours in the operating theater. Meanwhile, gynecology and obstetrics are considered women's fields. Child and adolescent psychiatry illustrates additional barriers. Few women pursue this path, and those who do often face opposition from their families and logistical obstacles to training abroad. One psychiatrist in training reflected:

*Sending their daughter to another country was itself a challenge for my parents and convincing them was another barrier. I wanted to encourage other women*.

Baijanthi, a psychiatry student from Nepal, is currently in India

Women physicians reported persistent inequities at work. They were more likely than their male colleagues to describe being stigmatized, having no access to maternity leave or private spaces for breastfeeding and limited institutional support during early career stages. Some observed that several women left medicine after graduation to become full‐time caregivers, highlighting the ongoing influence of household expectations and gendered divisions of labor.

These findings at the hospital level shed light on wider system hierarchies. Physicians occupy decision‐making positions that structure care pathways, supervision, referrals, and patient flow. Senior specialists have more balanced schedules and greater authority, whereas junior doctors have heavy workloads and limited control. In determining opportunity and recognition, gender intersects with seniority and specialization. This general trend is consistent with the broader care versus cure divide observed in other areas of the health system. District and tertiary hospitals are thus crucial training grounds, as well as places where professional hierarchies and gender norms are reproduced. This affects staff retention, employee well‐being, and the long‐term allocation of expertise within Nepal's medical workforce.

## 5 Discussion

In Nepal's healthcare sector, gender plays a significant role in all health professions and care institutions, whether centralized or decentralized. The results show that this tendency is exacerbated by gendered occupational segregation based on care and cure roles, as well as by gender norms and patriarchal societal dynamics (Bigler 2022; Ojha et al. 2025; Thieme et al. 2025). Across the three groups examined, women predominate: they form the overwhelming majority of FCHVs and nurses, and although more women are entering medicine, becoming a specialist remains challenging. Care professions are feminized and often entail voluntary work for FCHVs or low salaries for nurses, who receive only modest salary increases throughout their careers. These findings are supported by scholarly and policy debates about how feminized professions are linked to lower salaries and informal work (Carranza et al. 2019; Kabeer and Natali 2013).

Nepal's healthcare system relies heavily on the unpaid work of FCHVs, who contribute to SDG 3 by improving maternal and child health. This impact is well documented (Langer et al. 2015; Maher and Cometto 2016; Mehra et al. 2020), yet it rests on women's voluntary labor. Similarly, nurses at ward and hospital levels provide indispensable services that are persistently undervalued and have limited career progression opportunities. Both cases reflect Oswald Spring's (2020) and Mies and Shiva's (2016) argument that the current economic system depends on women's unpaid and unrecognized labor, as well as the exploitation of nature.

Gender norms create power imbalances, as exemplified by hierarchies in the health sector. Nurses working in decentralized settings, often without male colleagues, are less affected by gendered hierarchies and enjoy greater autonomy in their roles, which is linked with higher job satisfaction. Conversely, nurses in centralized settings frequently encounter gendered hierarchies that cause frustration and undermine decent working conditions (Chigbu and Nekhwevha 2023). Alongside expectations of better pay and conditions, this frustration drives migration to escape patriarchal structures. These gendered hierarchies also hinder the performance of health workers, affecting society at large and the long‐term sustainability of the health sector (Dossey et al. 2019).

The findings further demonstrate that power relations are shaped not only by gender, but also by age and years of experience. As physicians age and become more specialized, they benefit from higher pay and self‐determined schedules. Meanwhile, senior nurses hold authority over junior nurses, and residents face excessively long workdays and inadequate maternity protection, inconsistent with SDG 8 on decent work (UN 2015).

To achieve a gender‐equitable and sustainable health system, fundamental changes to discriminatory gender norms and power relations are needed through gender‐transformative approaches and gender‐responsive policies (Hay et al. 2019).

This study's strengths include its multiphase design across the three key groups within Nepal's health workforce, its participatory photovoice component, and the validation of findings with participants during the stakeholder meetings. As a qualitative study set in a specific, mostly urban context, its findings are not statistically generalizable but offer situated, contextual understanding.

## 6 Conclusion

The contributions of healthcare workers are vital for achieving sustainable development and meeting the objectives of the UN 2030 Agenda. Ensuring decent working conditions is essential to enable their full participation. Gender inequity undermines both decent work and societal sustainability. Analyzing Nepal's healthcare sector through the lenses of gender and decent work reveals that the current working environment is unsustainable and threatens the achievement of the SDGs. These dynamics reflect broader regional patterns: across South Asia, women dominate the health and care workforce. Nevertheless, they are concentrated in undervalued and low‐paid positions with limited prospects for advancement.

To promote sustainable growth in Nepal's public and private healthcare sectors and to meet SDG 3 (health), SDG 5 (gender equality), and SDG 8 (decent work), it is crucial to address gender equity as a means of achieving gender equality. A balanced power dynamic across all genders, professions, and age groups demands gender‐responsive reforms that challenge existing power structures and hierarchies. Addressing these issues is not only a national priority but also a critical step in advancing inclusive and sustainable development throughout South Asia.

## 7 Relevance for Clinical Practice and Policymakers

Drawing on all research phases, health workers identified the following practical priorities to advance decent work and sustainable health services (Table 3).

**TABLE 3:** Recommendations, evidence, actors, and implementation.

Recommendations	Supporting findings	Responsible actors	Implementation horizon
Gender equity in care delivery	Nurses' care ranked below doctors' cure and excluded from decisions (4.2), career stagnation regardless of qualification (4.2), and FCHVs covering their own travel (4.1)	Hospital and clinical leadership for recognition and advancement, health facilities and municipalities for transport costs, and federal labor and health policy for the formal recognition and valuation of care work	Recognition and cost coverage short term, advancement pathways medium term
Fair work, fair pay and social protection across all staff categories	Nurses' low pay and unpaid preemployment “volunteer” work (4.2), FCHV remuneration (4.1), and long hours and self‐sacrifice expectations (4.2, 4.3)	Federal labor and health ministries, employers, hospitals	Wage floors short to medium term, social security integration medium term
Training aligned with service needs and equitable access	FCHVs' requests for more frequent and mental‐health training (4.1), brain drain and aspirations to migrate abroad (4.3), and gendered barriers to women's advancement (4.3)	Health ministry and provinces, an independent education and workforce body (Medical Education Commission), training institutions	Training funding short to medium, enrolment regulation medium to long

*Note:* These three recommendations address one condition in different forms: the low value society ascribes to feminized care work. Recognizing it in daily practice, paying and protecting it as labor, and investing in training and advancement each confront the same devaluation, rooted in gender norms and the nonrecognition of social reproduction. Reversing it depends on policy and social norms, not on individual facilities alone.

### 7.1 Gender Equity in Care Delivery

Ensure that the value of care is both visible and appropriately rewarded. Acknowledge the role of nursing during daily rounds and case reviews. Establish pathways for advancement that recognize outstanding care. Cover transport and meal costs so that community follow‐up does not fall on staff.

### 7.2 Fair Work, Fair Pay, and Social Protection Across All Staff Categories

Establish wage floors for all essential positions to eliminate unpaid or underpaid work. Guarantee that overtime is compensated and rest periods between shifts are protected.

### 7.3 Training Aligned With Service Needs, and Equitable Access

Regularly fund competency‐based training for all groups, including community‐based workers, and ensure enrolment aligns with the system's capacity to absorb graduates. Ensure equal access for women to practical training, courses, and leadership development opportunities.

## Author Contributions

**Christine Bigler:** conceptualization, investigation, writing – original draft, methodology. **Sony K.C.:** conceptualization, investigation, writing – review and editing. **Susan Thieme:** conceptualization, investigation, funding acquisition, writing – review and editing, supervision.

## Funding

This research was funded by the University of Bern. The authors have nothing to report.

## Ethics Statement

This noninterventional interview study did not require formal ethics committee approval under the applicable institutional guidelines. All procedures complied with relevant ethical standards.

## Consent

All participants provided informed consent in writing prior to data collection, including consent to audio‐recording and the use of anonymized quotations.

## Conflicts of Interest

The authors declare no conflicts of interest.

## Data Availability

The data that support the findings of this study are available on request from the corresponding author. The data are not publicly available due to privacy or ethical restrictions.

## Endnotes

^1^
This paper primarily refers to women and men, reflecting the binary gender categories used in the available data and by participants. It acknowledges, however, that gender is nonbinary and includes diverse identities and experiences beyond this dichotomy (Butler 1990; West and Zimmerman 1987).

^2^
The WHO defines gender equity as the process of ensuring fairness by compensating for historical and social disadvantages so that all genders can achieve equal health and work outcomes. Equity is the process, and gender equality is the outcome (UN Women 2022; WHO 2019).

^3^
This partly resonates with a recent study showing that the proportion of women among medical graduates in Nepal is increasing. While not up to 60%, it has risen from 8.1% before 2000 to 37.4% after 2020 (Ojha et al. 2025).
